# Clinical Spectrum, Microbiological Characteristics, and Outcomes of Apophysomyces Infections in Immunocompetent Patients: A Case Series From North India

**DOI:** 10.7759/cureus.113817

**Published:** 2026-08-02

**Authors:** Disha Gautam, Malini Capoor, Moumita Chakraborty, Neena Chaudhary, Sujata Sarabahi, Chandan Kumar R, Shrikrishna Parab, Ishapreet Tuli

**Affiliations:** 1 Clinical Microbiology, Vardhman Mahavir Medical College & Safdarjung Hospital, New Delhi, IND; 2 Otolaryngology - Head and Neck Surgery, Vardhman Mahavir Medical College & Safdarjung Hospital, New Delhi, IND; 3 Burns and Plastic Surgery, Vardhman Mahavir Medical College & Safdarjung Hospital, New Delhi, IND

**Keywords:** apophysomyces, cutaneous mucormycosis, immunocompetent host, india, mucormycosis, trauma

## Abstract

Background: *Apophysomyces* species are emerging etiological agents of mucormycosis, particularly in tropical and subtropical regions. Unlike classical mucormycosis, which predominantly affects immunocompromised individuals, *Apophysomyces* infections frequently occur in immunocompetent hosts following traumatic implantation of fungal spores.

Objectives: To describe the clinical manifestations, microbiological characteristics, management strategies, and outcomes of *Apophysomyces* infections diagnosed at a tertiary care center in North India.

Methods: A retrospective observational study was conducted on patients with culture-confirmed and molecularly confirmed *Apophysomyces* infections diagnosed between January 2019 and December 2024. Demographic details, predisposing factors, clinical presentation, laboratory findings, treatment modalities, and outcomes were reviewed.

Results: We reported 10 (25%) cases of primary cutaneous, rhino-orbital, pulmonary mucormycosis due to *Apophysomyces *sppamong 40 cases of mucormycosis diagnosed during the study period. On direct microscopy and culture, the isolates were identified as *Apophysomyces variabilis (A. variabilis) *in nine cases (90%) and *Apophysomyces elegans (A. elegans)* in one case (10%). The mean age of patients was 39.5 years (range: 1 month-60 years), and males were predominant, that is, seven cases (70%). Trauma-associated inoculation was the predominant predisposing factor, accounting for the majority of infections. Clinical manifestations included osteomyelitis, necrotizing fasciitis, cutaneous and subcutaneous infections, chest wall involvement, postoperative wound infection, and rhino-orbital disease. Extensive surgical debridement and medical management led to the recovery of six cases (60%), whereas four (40%) succumbed due to a delay in approaching our centre. Species identification was confirmed by internal transcribed spacer (ITS) and 28S recombinant DNA (rDNA) sequencing. Combined surgical debridement and liposomal amphotericin B therapy was associated with improved outcomes.

Conclusions: *Apophysomyces* species represent an emerging cause of invasive mucormycosis among immunocompetent individuals in North India. Trauma remains the principal predisposing factor. Early microbiological diagnosis, prompt surgical intervention, and timely antifungal therapy are critical determinants of favourable clinical outcomes.

## Introduction

Mucormycosis is a rapidly progressive and potentially fatal angioinvasive fungal infection caused by fungi belonging to the order Mucorales. Despite advances in diagnostic modalities and antifungal therapy, mucormycosis remains associated with substantial morbidity and mortality, particularly in patients with delayed diagnosis or extensive disease involvement. The infection is most commonly caused by species of *Rhizopus, Mucor, Lichtheimia,* and *Rhizomucor*, although infections caused by less frequently encountered Mucorales are increasingly being recognized worldwide [1,2]. The genus *Apophysomyces* includes six species; only *Apophysomyces elegans* (*A. elegans), Apophysomyces​​​​​​​ mexicanus (A. mexicanus), Apophysomyces* *ossiformis (A. ossiformis)*, and *Apophysomyces​​​​​​​ variabilis* (*A. variabilis) *have been reported to cause infections in both immunocompetent and immunocompromised patients. *A. elegans* was isolated from soil of a mango orchard in India. Following the initial description of *A. elegans*, numerous case reports and small case series have documented infections caused by *Apophysomyces* species worldwide. However, comprehensive molecularly confirmed case series from North India remain scarce. The present study represents one of the first detailed reviews of *Apophysomyces* infections from this region, highlighting the clinical characteristics, risk factors, microbiological diagnosis, management, and outcomes.

*Apophysomyces* species have been isolated from soil in several tropical and subtropical regions, including India, Australia, Brazil, and the United States, supporting their environmental distribution and association with traumatic inoculation. Traditionally, mucormycosis has been associated with diabetes mellitus, hematological malignancies, solid organ transplantation, prolonged corticosteroid therapy, and other forms of immunosuppression. In contrast, *Apophysomyces* infections frequently occur in immunocompetent individuals, particularly following trauma or environmental exposure, making early clinical suspicion and prompt microbiological diagnosis essential for improving patient outcomes [1-4].

Among the emerging etiological agents of mucormycosis, *Apophysomyces* species have gained considerable clinical significance because of their distinctive epidemiological and pathogenic characteristics. Unlike classical mucormycosis, which predominantly affects immunocompromised individuals, *Apophysomyces* infections frequently occur in otherwise healthy and immunocompetent hosts [5,6]. Traumatic implantation of fungal spores through contaminated soil, vegetation, or environmental debris into disrupted skin and soft tissues is considered the principal mode of acquisition [5-8]. Consequently, these infections commonly manifest as cutaneous and subcutaneous disease, necrotizing fasciitis, osteomyelitis, deep soft-tissue infections, and, less frequently, rhino-orbital or disseminated disease [6-9].

India is recognized as one of the global hotspots for mucormycosis, with prevalence estimates substantially higher than those reported from developed countries [3,10,11]. Several studies have highlighted the relatively high occurrence of *Apophysomyces* infections in tropical and subtropical regions, particularly following trauma-related injuries, road traffic accidents, and natural disasters [6,9,12]. The ability of these fungi to grow at elevated temperatures and invade vascular structures contributes to their aggressive clinical behaviour and poor outcomes when diagnosis and treatment are delayed [5,13].

Laboratory diagnosis of *Apophysomyces* infections remains challenging because the organism often exhibits poor sporulation on routine mycological media, resulting in delayed or inaccurate identification [8,14]. While direct microscopy and culture remain important diagnostic tools, molecular methods, such as internal transcribed spacer (ITS) and 28S ribosomal DNA sequencing, have significantly improved species-level identification and diagnostic accuracy [14,15]. Early recognition, prompt surgical debridement, and timely initiation of antifungal therapy, particularly liposomal amphotericin B, are essential for improving clinical outcomes [16].

Despite increasing recognition of *Apophysomyces* as an important cause of invasive mucormycosis, data describing its clinicomicrobiological spectrum and outcomes from North India remain limited. The present study describes the clinical characteristics, microbiological findings, treatment approaches, and outcomes of ten culture-confirmed and molecularly confirmed cases of *Apophysomyces *infection diagnosed at a tertiary care center in North India over a six-year period.

## Materials and methods

Study design and setting

A retrospective observational study was conducted at a tertiary care teaching hospital in North India. All patients diagnosed with *Apophysomyces* spp infection between January 2019 and December 2024 were reviewed.

Case definition and inclusion criteria

Patients were included if they had clinically suspected mucormycosis with microbiological evidence of *Apophysomyces *species demonstrated by fungal culture and confirmed by molecular identification. Patients with incomplete microbiological records or without culture confirmation were excluded.

Data collection

Demographic characteristics, underlying conditions, predisposing factors, clinical manifestations, laboratory findings, treatment modalities, and clinical outcomes were extracted from hospital records. Particular attention was given to the presence of trauma, surgical procedures, immunosuppressive conditions, and the anatomical site of infection.

Predisposing factors

Trauma-associated inoculation was identified as the most frequent predisposing factor. Road traffic accidents, blunt facial injuries, postoperative wound complications, and soft tissue trauma accounted for the majority of cases. Eight patients (80%) had no identifiable immunocompromising condition. One patient was a premature neonate, and another had a history of pulmonary tuberculosis. The suspected cases of invasive rhinosinusitis were found to be infected by *Apophysomyces *species and were tested for COVID-19 as an important risk factor, but all patients tested negative for COVID-19 infection. None of the cases were reported as COVID-19-infected, but as an outbreak during 2020, other species such as *Rhizopus, Mucor, and Rhizomucor* spp. from rhinosinus samples were reported.

Clinical manifestations

The clinical spectrum was heterogeneous and included osteomyelitis (n=1), necrotizing fasciitis (n=1), rhino-orbital mucormycosis (n=1), postoperative wound infection (n=1), chest wall infection (n=1), and various forms of cutaneous and subcutaneous disease (n=5). Extensive soft tissue involvement and tissue necrosis were common findings among patients presenting after trauma. 

Mycological investigations

Clinical specimens, including tissue biopsies, debrided tissue, wound exudates, pus, and respiratory samples, were processed according to standard mycological procedures. Direct microscopic examination was performed using 10% potassium hydroxide (KOH) wet mounts. The presence of broad, ribbon-like, pauciseptate or aseptate hyphae was considered suggestive of mucormycosis. Samples were cultured on Sabouraud's dextrose agar and incubated at 25°C and 37°C. Isolates demonstrating rapid growth with characteristic fluffy, cottony colonies were subjected to further microscopic examination using lactophenol cotton blue mounts (LPCB) and slide culture preparations. Morphological identification was based on the presence of elongated sporangiophores, bell-shaped apophyses, pyriform sporangia, and rhizoids arising opposite the sporangiophores. On the slide culture, there were long sporangiophores up to 500 µm, along with half circle columella. Sporangia are pear-shaped or pyriform. The growth was resistant to cycloheximide. These findings were conclusive of *A. elegans*.

Molecular identification

Species confirmation was performed using sequencing of the internal transcribed spacer (ITS) region and D1/D2 domains of the 28S large ribosomal DNA, and one out of 10 cases (10%) was *A. elegans*, and nine out of 10 cases (90%) were confirmed as *A. variabilis*. Sequence data were compared with reference sequences available in GenBank using the BLAST algorithm. The confirmation was done at the National Culture Collection of Pathogenic Fungi, at the Post Graduate Institute of Medical Education and Research (PGIMER), Chandigarh, India. Antifungal susceptibility was not performed for these isolates.

Statistical analysis

Descriptive statistics were used to summarize the data. Continuous variables are presented as means and ranges, while categorical variables are presented as frequencies and percentages.

## Results

**Figure 1:**
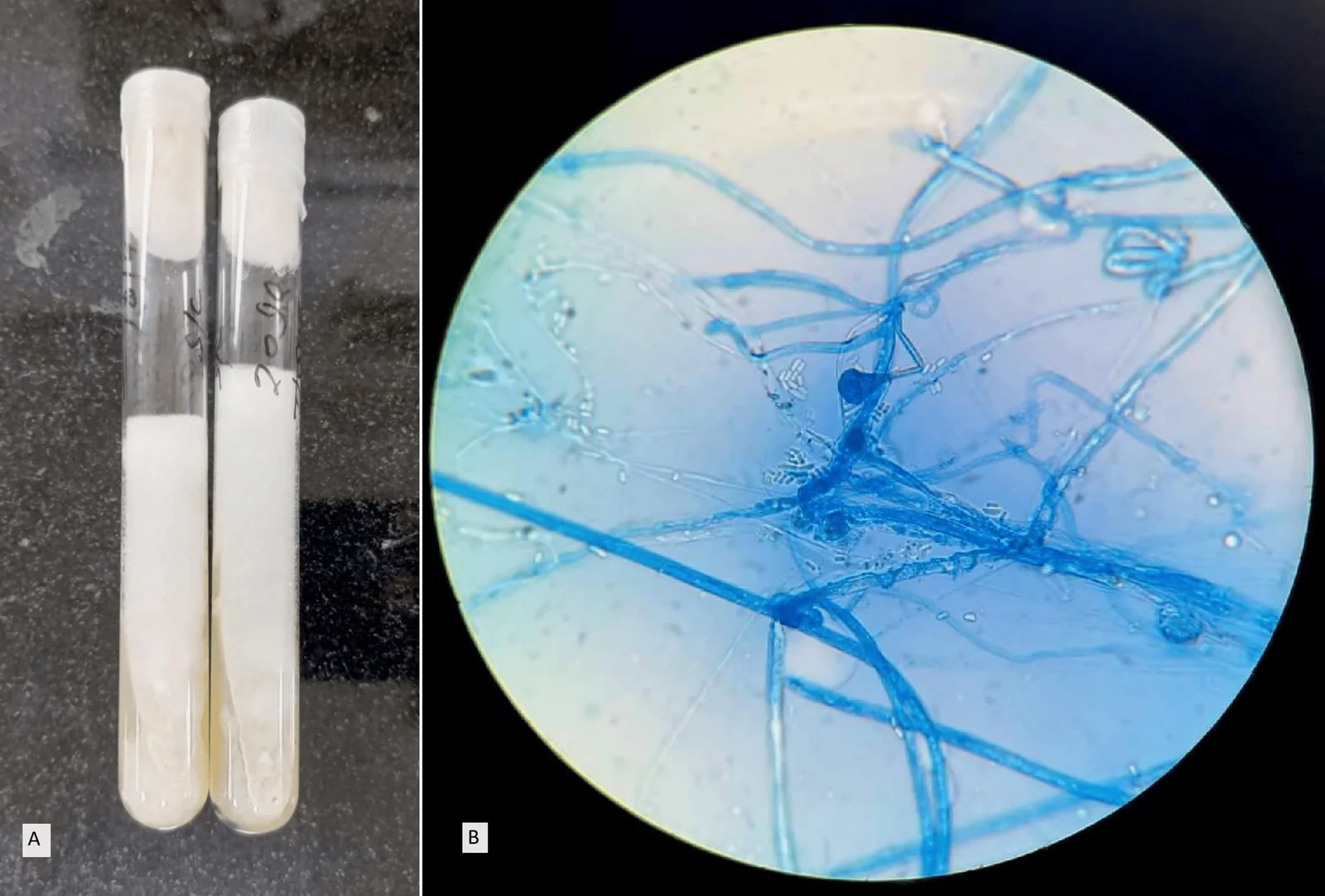
(A) Cottony, fluffy growth of Apophysomyces species filling Sabouraud dextrose agar tube. (B) Lactophenol cotton blue mount showing elongated sporangiophores with bell-shaped apophyses, pyriform sporangia, and rhizoids opposite sporangiophores.

During the six-year study period, 40 patients were diagnosed with mucormycosis, of whom 10 (25%) had culture-confirmed and molecularly confirmed *Apophysomyces* infection. The isolates demonstrated rapid growth with characteristic fluffy, cottony colonies (Figure 1A), and the slide culture showed elongated sporangiophores, bell-shaped apophyses, pyriform sporangia, and rhizoids arising opposite the sporangiophores (Figure 1B). On the slide culture, there were long sporangiophores up to 500 µm, along with half circle columella, suggesting the findings as *Apophysomyces *species (Figure 1).

The cases were often associated with traumatic inoculation, such as road traffic accidents, post-operative wounds, and necrotizing fasciitis. The isolates were identified predominantly as *A. variabilis*, consisting of nine cases (90%), and *A. elegans *was detected in one case (10%). Trauma-associated inoculation was identified as the most frequent predisposing factor. Eight patients (80%) had no identifiable immunocompromising condition. One patient was a premature neonate, and another had a history of pulmonary tuberculosis. Clinical presentations ranged from cutaneous and subcutaneous infections to rhino-orbital involvement. Despite aggressive treatment, the mortality rate was 40%, with four deaths among 10 cases. The study population comprised seven males (70%) and three females (30%). Patient age ranged from one month to 60 years, with a mean age of 39.5 years. The cases included the youngest patient, a one-month-old female (premature birth), and the oldest patient, 60 years of age. The morbidity, including rhino-orbital involvement, necrotizing fasciitis, or osteomyelitis, had worse outcomes, whereas the one-month-old infant had severe facial involvement and succumbed to death (six out of 10 patients survived). Early surgical debridement combined with liposomal amphotericin B therapy was associated with improved clinical outcomes. Facial or post-traumatic mucormycosis with timely intervention improved, whereas systemic involvement, especially in infants or immunocompromised patients, correlates with higher mortality. Nine (90%) patients received liposomal amphotericin B, while one patient received antibacterial therapy alone before the fungal etiology was recognized. Surgical debridement was performed in six (60%) patients. Combined surgical and antifungal treatment was associated with improved clinical outcomes. Six patients survived, resulting in an overall survival rate of 60%, whereas four patients died, yielding an overall mortality rate of 40%. Mortality was observed primarily among patients with extensive disease, delayed presentation, or severe underlying clinical conditions (Table 1).

**Table 1:** Review of suspected mucormycosis cases diagnosed between 2019 and 2024 at a tertiary care hospital in North India.

Case	Age (yrs)	Gender	Predisposing factors	Clinical features	Sample	Treatment	Outcome
1.	35 yrs	Male	None	Osteomyelitis, post road traffic accident, fever	Drainage of the pus	Liposomal amphotericin B and antibiotics	Patient survived
2.	32 yrs	Male	None	Necrotizing fasciitis over the abdominal wall, thighs, and scrotum; diagnosed as Meleny’s gangrene; fever	Debridement of the affected area	Liposomal amphotericin B and antibiotics	Patient expired
3.	1 month	Female	Prematurity (born at 7^th^ month)	Difficulty in breathing, eschar over the right side of the face, facial swelling, fever	Aspirated pus	Liposomal amphotericin B	Patient expired
4.	40 yrs	Male	None	Fracture of the right tibia post road traffic accident	Debridement of the affected area	Liposomal amphotericin B	Patient expired
5.	36 yrs	Male	None	Facial swelling, fever	Debridement of the affected area	Liposomal amphotericin B	Patient survived
6.	60 yrs	Male	None	Right facial and eye swelling, nasal obstruction, proptosis	Debridement of the affected area	Liposomal amphotericin B and antibiotics	Patient survived
7.	27 yrs	Male	None	Blunt facial trauma, road traffic accident	Debridement of the affected area	Liposomal amphotericin B	Patient survived
8.	52 yrs	Female	None	Post-operative wound dehiscence, midline non-healing wound	Debridement of the affected area	Liposomal amphotericin B	Patient survived
9.	60 yrs	Male	None	Swelling and pain over the right chest wall, itching, foul-smelling discharge with skin discolouration to blackish necrotic patches	Debridement of the affected area	Liposomal amphotericin B	Patient survived
10.	52 yrs	Female	Tuberculosis	Cough, fever, shortness of breath	Endotracheal aspirate	Antibiotics	Patient expired

## Discussion

The present study describes 10 cases of *Apophysomyces* infection diagnosed over a six-year period at a tertiary care center in North India and highlights the emerging importance of this pathogen as a cause of invasive mucormycosis among immunocompetent individuals. Unlike classical mucormycosis, which is predominantly associated with diabetes mellitus, hematological malignancies, organ transplantation, and corticosteroid exposure, *Apophysomyces* infections frequently occur in otherwise healthy individuals [17,18]. In our series, 80% of patients lacked traditional risk factors for invasive fungal disease, supporting previous observations that traumatic inoculation plays a pivotal role in disease pathogenesis [19,20].

Infection with *A. elegans* is mainly the result of the inoculation of soil or plant debris during an accident. Well-described causes of traumatic inoculation are motorcycle and other road traffic accidents, similar to our cases, where three of the cases came with a history of road traffic accidents. Although inhalation of *A. elegans* spores can result in rhinocerebral and pulmonary infections, local wound contamination occurred in our other four cases. The isolates were identified as *A. elegans* (one) and *A. variabilis *(nine) in our study, and trauma-associated inoculation was identified as the most frequent predisposing factor. The majority of involved patients demonstrated no underlying immune system dysfunction in our review. Trauma-related infection represented the predominant mode of acquisition in the present study. Similar findings have been reported from India and other tropical regions, where contaminated soil and environmental debris introduced into wounds following road traffic accidents or penetrating injuries facilitate fungal invasion [19,21]. The ability of *Apophysomyces* species to grow at elevated temperatures and rapidly invade soft tissues contributes to their aggressive clinical behaviour [18].

Cutaneous and subcutaneous infections represented the most common presentation in our cohort. However, severe manifestations, including necrotizing fasciitis, osteomyelitis, chest wall infection, and rhino-orbital disease, were also encountered. These observations are consistent with previous reports describing a broad spectrum of disease ranging from localized cutaneous lesions to life-threatening invasive infections [20-22]. *Apophysomyces* infections do not have a high death rate; unlike in our case review, we reported 40% mortality, which was associated with its occurrence in immunocompetent patients, which is again reported rarely.

Laboratory diagnosis remains challenging. *Apophysomyces *species frequently fail to sporulate on routine laboratory media, leading to delays in identification. In the present study, definitive diagnosis required a combination of direct microscopy, culture, morphological characterization, and molecular sequencing. The incorporation of molecular methods significantly improved diagnostic confidence and enabled accurate species identification [18,23].

Combined surgical debridement and antifungal therapy remain the cornerstone of management. Most survivors in our series underwent aggressive surgical intervention together with liposomal amphotericin B administration. Previous studies have similarly demonstrated improved outcomes when extensive debridement is performed early in the course of disease [17,20,24].

A notable finding of the present study was the overall mortality rate of 40%. This mortality is considerably higher than that reported for localized primary cutaneous *Apophysomyces* infections but is comparable to rates observed in patients with deep tissue invasion, necrotizing fasciitis, rhino-orbital involvement, or delayed diagnosis. Chander et al. reported particularly poor outcomes among patients with *A. variabilis*-associated necrotizing soft tissue infections, where only one of five patients survived despite antifungal therapy and surgical intervention, emphasizing the aggressive nature of advanced disease [21]. Similarly, recent systematic reviews of invasive *Apophysomyces* infections have demonstrated that mortality increases substantially once infection extends beyond cutaneous tissues or when diagnosis is delayed [19]. The 40% mortality observed in our cohort likely reflects the presence of severe manifestations, including necrotizing fasciitis, osteomyelitis, rhino-orbital disease, and extensive trauma-associated infections. Furthermore, several patients were referred late to our tertiary-care center after significant disease progression, highlighting the importance of early clinical suspicion, rapid microbiological diagnosis, and prompt initiation of combined surgical and antifungal management.

The predominance of *A. variabilis* (90%) in our series is particularly noteworthy and is consistent with recent observations from northern India. Although *A. elegans* was historically considered the principal pathogenic species, advances in molecular taxonomy have demonstrated that many earlier isolates were misclassified because species-level identification was based solely on morphology. Molecular studies from India have subsequently shown that *A. variabilis* is now the most frequently recovered species, similar to our case series from human infections, especially necrotizing cutaneous and soft tissue infections occurring in immunocompetent individuals [17]. The predominance of *A. variabilis* in North India may be related to ecological adaptation of the species to the tropical and subtropical climate, its documented abundance in soil and environmental reservoirs, and improved molecular diagnostic techniques that allow accurate differentiation within the *Apophysomyces* complex [17,18]. In addition, India accounts for the majority of reported global *Apophysomyces* infections, suggesting that specific environmental and climatic conditions may favor persistence and transmission of *A. variabilis* in this region. Our findings further strengthen the growing evidence that *A. variabilis* has emerged as the dominant pathogenic species of the genus in northern India [20,21].

This series represents one of the largest molecularly confirmed collections of *Apophysomyces* infections reported from North India. Better outcomes were likely attributable to early diagnosis, prompt surgical debridement, and timely antifungal therapy. The findings emphasize that invasive apophysomycosis should be considered even in immunocompetent patients presenting with post-traumatic soft tissue infections, highlighting the importance of early mycological investigation and multidisciplinary management.

The present study is limited by its retrospective design, single-center setting, and relatively small sample size, which may affect the generalizability of the findings. As *Apophysomyces* infections are uncommon, the number of cases was insufficient to perform comparative analyses of predictors of mortality or treatment outcomes. There was an absence of antifungal susceptibility testing, limited methodological detail for molecular identification, or the inability to infer treatment effectiveness from the descriptive case series. Additionally, incomplete long-term follow-up data prevented assessment of recurrence rates and functional outcomes following treatment. Despite these limitations, this case series represents one of the largest molecularly confirmed collections of* Apophysomyces *infections reported from North India and provides clinically relevant insights into their presentation, diagnosis, management, and outcomes.

## Conclusions

*Apophysomyces *species are increasingly recognized as important etiological agents of mucormycosis in tropical countries, particularly among immunocompetent individuals following trauma. The present case series highlights the diverse clinical manifestations and substantial mortality associated with these infections. Though this descriptive design does not permit causal inference, early clinical suspicion, prompt microbiological diagnosis, aggressive surgical debridement, and timely antifungal therapy remain essential for improving patient outcomes. Greater awareness among clinicians and microbiologists is required to facilitate early recognition and effective management of this emerging pathogen.
